# An MCBJ case study: The influence of π-conjugation on the single-molecule conductance at a solid/liquid interface

**DOI:** 10.3762/bjnano.2.76

**Published:** 2011-10-18

**Authors:** Wenjing Hong, Hennie Valkenier, Gábor Mészáros, David Zsolt Manrique, Artem Mishchenko, Alexander Putz, Pavel Moreno García, Colin J Lambert, Jan C Hummelen, Thomas Wandlowski

**Affiliations:** 1 Department of Chemistry and Biochemistry, University of Bern, Freiesstrasse 3, CH-3012, Bern, Switzerland; 2 Stratingh Institute for Chemistry and Zernike Institute for Advanced Materials, University of Groningen, Nijenborgh 4, 9747 AG Groningen, The Netherlands; 3 Institute of Materials and Environmental Chemistry, Chemical Research Centre, Hungarian Academy of Sciences, Pusztaszeriút 59-67, H-1025 Budapest, Hungary; 4 Lancaster University, Department of Physics, Lancaster LA1 4YB, England; 5 Institute of Bio- and Nanosystems IBN 3 and Center of Nanoelectronic Systems for Informational Technology, Research Center Juelich, D-52425 Juelich, Germany

**Keywords:** anthraquinone, π-conjugation, mechanically controlled break junction, single-molecule conductance

## Abstract

π-Conjugation plays an important role in charge transport through single molecular junctions. We describe in this paper the construction of a mechanically controlled break-junction setup (MCBJ) equipped with a highly sensitive log *I–V* converter in order to measure ultralow conductances of molecular rods trapped between two gold leads. The current resolution of the setup reaches down to 10 fA. We report single-molecule conductance measurements of an anthracene-based linearly conjugated molecule (**AC**), of an anthraquinone-based cross-conjugated molecule (**AQ**), and of a dihydroanthracene-based molecule (**AH**) with a broken conjugation. The quantitative analysis of complementary current–distance and current–voltage measurements revealed details of the influence of π-conjugation on the single-molecule conductance.

## Introduction

Molecular electronics has expanded tremendously during the past ten years [1–13]. A comprehensive understanding of charge transport through single molecules and tailored nanojunctions is a fundamental requirement for further electronic-circuit and device design. For instance, the role of length [14–15] and molecular conformation [13,16] and as well as of the anchoring group and of the contacting leads [17–18] was studied to develop correlations between charge-transport characteristics and molecular structure. Furthermore, π-conjugation plays an essential role in charge transport through single molecular junctions, and has attracted great interest in organic synthesis [19–20], conductance measurements [1,3,8–10,16,21–23] as well as in theoretical calculations [1,24–25]. In particular, single-molecule conductance measurements provide direct access to unravel the influence of π-conjugation on the molecular (-junction) conductance. However, due to the relative low conductance of broken-conjugated and cross-conjugated rigid rodlike molecules [26], reliable transport measurements through these types of molecular junctions are still a challenging topic.

Charge-transport characteristics of single molecules or of a few molecules trapped between two leads were studied in various experimental platforms. These include scanning tunneling microscopy (STM) [27–29], current probe atomic force microscopy (CP-AFM) [30–32], scanning tunneling spectroscopy (STS) or STM-break junction (STM-BJ) [13,15–16,33–36], crossed-wire geometry [37], nanoparticle junctions [38–39], mechanically controlled break junctions (MCBJ) [40–45], electromigration setups [46–47], nanopores [48], and liquid metal junctions employing mercury [49–50] or eutectic alloys of gallium and indium (EGaIn) [51].

STM-BJ and MCBJ are the two most popular and reliable approaches for single-molecule conductance measurements. Reed et al. [40], Kergueris et al. [41], Reichert et al. [42] and Smit et al. [43] pioneered the MCBJ technique to measure charge transport through single molecules. Xu et al. developed an STM-BJ technique based on the formation and breaking of thousands of individual molecular junctions by repeatedly approaching and withdrawing a STM tip towards and away from a substrate in the presence of sample molecules [34]. The MCBJ technique, as compared with the STM-BJ approach, allows control of the separation between two electrodes with extremely high stability and precision [52], which attracted great interest with respect to its application in molecular charge-transport studies [40–45].

**Figure 1:**
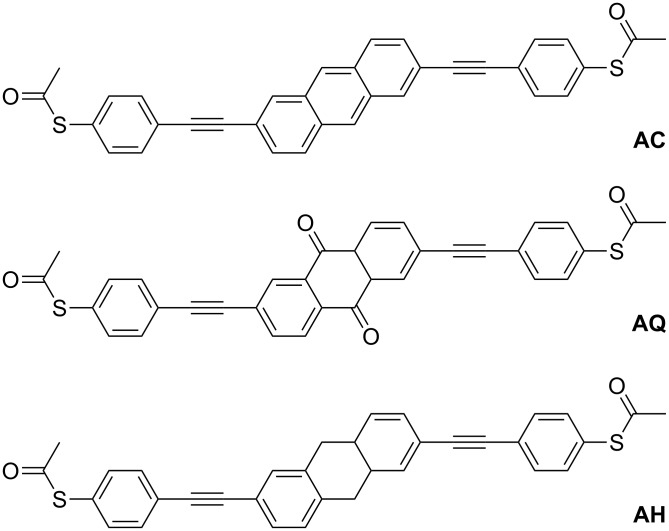
Molecular structures of **AC**, **AQ**, and **AH**.

In the present paper we explore the influence of π-conjugation on the conductance of single-molecule junctions of oligophenylene ethynylene (OPE)-type molecules contacted to gold leads. We have chosen three rigid dithiolated molecular wires with different conjugation patterns: An anthracene-based linearly conjugated wire (**AC**), an anthraquinone-based cross-conjugated wire (**AQ**), and a dihydroanthracene-based wire with a broken π-conjugation (**AH**) (Figure 1).

The transport characteristics in single molecular junctions were investigated by conductance–distance as well as continuous current–voltage measurements in complementary MCBJ and STM-BJ experiments. In particular, a custom-designed MCBJ setup was equipped with a high-sensitivity logarithmic *I–V* converter [53] , which enabled current measurements down to 10 fA with a high dynamic range.

The paper is organized as follows: We will first introduce our novel instrumental and methodological developments, and we shall subsequently focus on one case study. We present quantitative MCBJ experiments of a family of custom-designed OPE-type rigid molecular rods at a solid/liquid interface. In particular, we will address the influence of π-conjugation on the single-junction conductance.

## Experimental

### System configuration

The MCBJ technique provides a high mechanical stability [52] due to the short distance between the two free-standing electrode-tip ends and the support. In consequence, molecular junction stretching and formation processes can be controlled with high precision and stability on the time scale of seconds, even at room temperature and in solution. The construction of an “ideal” platform for charge-transport measurements of single molecular junctions at solid/liquid interfaces requires the consideration of the following key factors: The variation of the conductance in different types of single-molecule systems, as well as the tunneling decay in the subnanometer scale demands precise current measurements in a high dynamic range, from microamperes (μA) down to a few femtoamperes (fA). Moreover, the current changes over five to eight orders of magnitude in a few milliseconds, which requires a fast response in the current measurements.

The second requirement relates to the motion control of the pushing rod. The pushing rod bends the sample substrate, thus enabling the adjustment of the distance between the two gold electrodes (attenuation ratio ~0.01). The pushing distance reaches several hundreds of micrometers, while the resolution is controlled at the subnanometer level. Experiments with notched-wire samples show a characteristic displacement ratio between the vertical (pushing rod) and the horizontal (nanoscale gap between the leads) movement of about 0.01. Lithographically prepared samples were reported with displacement ratios ranging between 10^−4^ to 10^−6^ [54]. On the other hand, notched gold-wire samples with a typical displacement ratio of 0.01 are rather sensitive to mechanical vibrations, which could interfere with the exact horizontal adjustment of the distance between the two electrodes. As a consequence, mechanical vibration due to the movement of the pushing rod should be minimized as much as possible.

**Figure 2:**
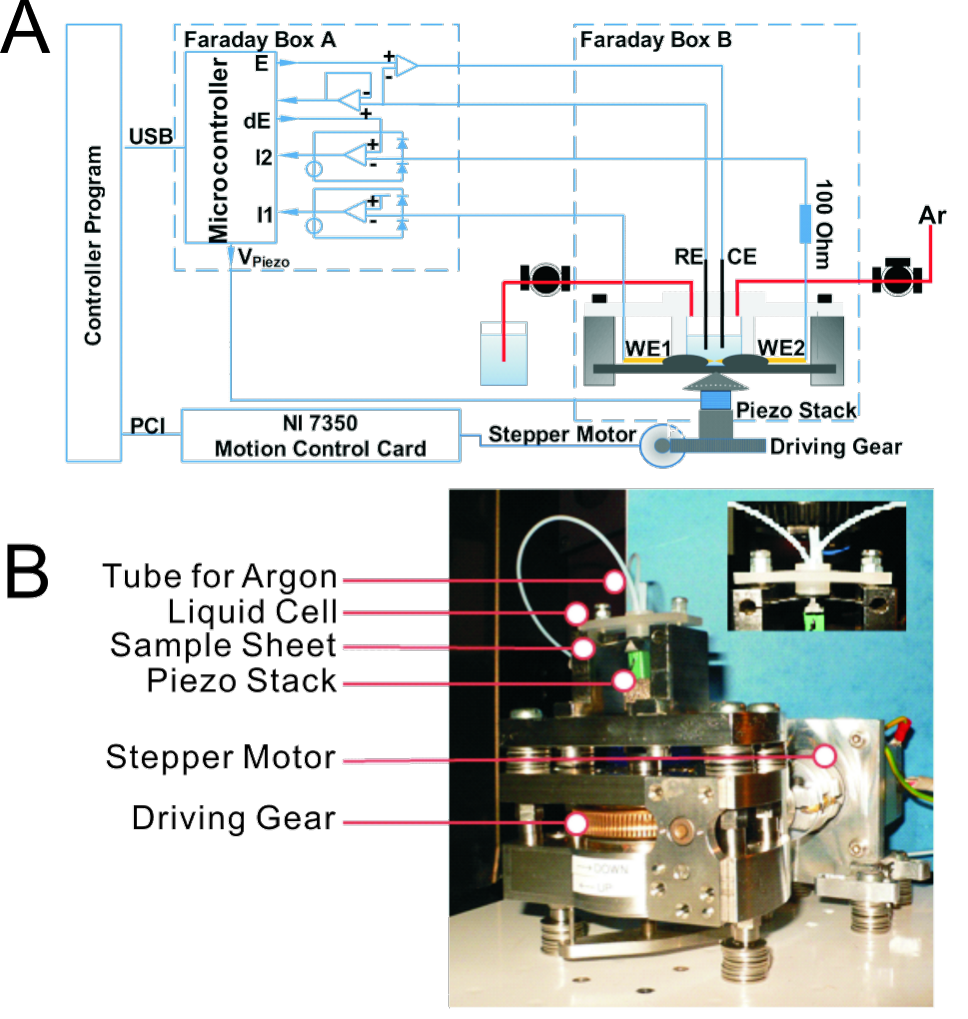
(A) Schematics of the system configuration and (B) pictures of the mechanical part in the MCBJ setup. The inset shows a detailed view of the liquid cell including the sample holder. For clarity, the Faraday shielding boxes were removed.

Thirdly, single-molecule measurements are often rather sensitive to the ambient environment, in particular to oxygen and to light. As a consequence, a closed liquid cell with inert gas protection and a continuous liquid flow is also needed. To match these three requirements, we constructed a MCBJ setup with a logarithmic *I–V* converter and implemented the *z*-movement of the pushing rod by combining a piezo stack and a stepper motor. Both design principles ensured a highly dynamic and precise current measurement, a long-distance *z*-movement, and subnanometer resolution. The implemented liquid cell has a filling volume of 150 μL. A tubing system for inert solution exchange and gas purging is also attached (Figure 2).

### Electronics design

#### Controller and current-measurement units

The MCBJ controller is based on a laboratory-built bipotentiostat. Two custom-designed bipolar and tunable logarithmic *I–V* converters [53] were implemented for measuring the current of the two gold leads labeled as working electrodes WE1 and WE2. The reference electrode RE and the counter electrode CE serve to control the potential. The driving signal of the piezo stack is supplied by an additional A/D-converter output of the controlling unit. The setup also permits the implementation of advanced functions during measurements with various trigger options, such as multistep, stop-and-hold movements or more complex modulations of the vertical *z*-displacement.

Buffered data acquisition and all timing-sensitive functions are performed directly by the onboard trigger operations of the microcontroller. The PC attached serves only as the user interface. The communication through an opto-isolated USB interface proceeds with a sampling rate of up to 12.5 kHz for the simultaneous recording of three data channels.

The controller unit provides three analog control signals. The first one controls the potential of WE1, which is particularly important for advanced electrochemical experiments with the MCBJ setup. The second one controls the voltage difference between the two working electrodes WE1 and WE2 (bias voltage), which drives the current through the two gold electrodes for the conductance measurements. The third channel controls the voltage output for the piezo stack in the range of 0 to 50 V allowing the displacement of the piezo stack down to 10 μm.

The stable and precise operation of the logarithmic *I–V* converter over a wide dynamic current range requires strict temperature control. In order to avoid any interference with the temperature-control unit we applied an analog PID controller with diodes as heating elements, which kept the temperature of the current-sensing diodes of the logarithmic *I–V* converter within ±0.05 K.

#### Noise control and electronics shielding

Two metallic Faraday boxes are used, one for the mechanical unit and the other for the controller unit (c.f. Figure 2) in order to avoid electronic cross talk between the different functional parts of the setup. The two electrodes of the MCBJ setup are connected to the controller through special low-noise coaxial cables. Operation of the stepper motor introduces considerable noise. As a consequence and to avoid this kind of interference, the stepper motor is placed outside the Faraday box. Furthermore, the stepper motor is used only for the coarse approach, and then switched off during the actual measurements, leaving only the piezo actuator in operation. The rotating coarse motion is transferred through a drive bearing through the hole in the shielding box to the pushing rod. This leads to an assembly of the mechanical unit with the piezo stack being the only electronic component inside the shielding box of the mechanical unit. In order to reduce possible electrical interference, the piezo stack is shielded with an additional compartment constructed from metalized-plastic fabrics. All shielding parts are connected to ground.

### Motion control

The motion control of the MCBJ set up is based on the combination of a stepper motor (Accu-coder 95511 from Encoder Production) with a piezo stack on top. The moving distance is 17 μm for a voltage range of 110 V. Typically we applied a voltage between 0 and 50 V. The mechanical part of the MCBJ is positioned on a vibration-isolation breadboard (Newport RG Breadboard), which is mounted on a passive granite table to further decrease the interference from ambient mechanical vibrations and shock waves.

The tunneling current between the two working electrodes WE1 and WE2 at a given bias voltage, the latter typically ranging from 0.020 V to 0.200 V, is chosen as the feedback signal. The pushing process starts with the stepper motor. Once a current decrease is detected, which represents the breaking of the gold–gold contact, the stepper motor is paused, and the *z*-motion control is switched to the piezo stack. The pushing rod is subsequently only driven by the application of a voltage to the piezo stack, which is ramped at a preset rate (between 0.01 V·s^−1^ and 25 V·s^−1^).

**Figure 3:**
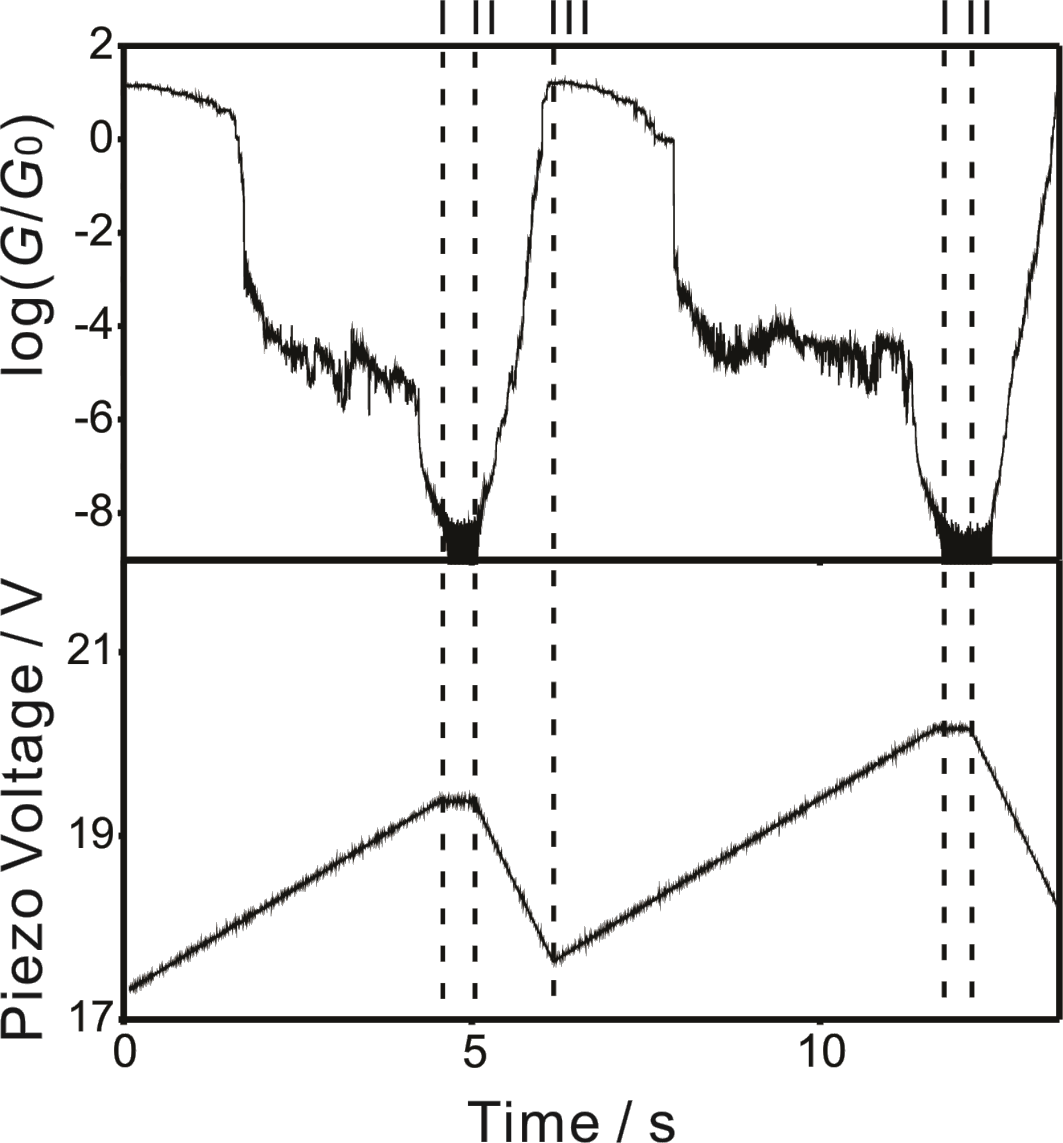
Conductance and voltage output for the piezo stack versus time for 0.1 mM **AC** in THF/decane (v:v = 1:4) under an Ar atmosphere at 0.10 V bias voltage.

The voltage output for the piezo stack communicates with an onboard trigger. The trigger senses the tunneling current, which is converted to the respective conductance. If the conductance reaches the noise threshold (*G* < 10^−8^* G*_0_; dashed line I in Figure 3), the voltage ramp for the piezo stack stops and after a preset waiting time (typically 0.5 s; dashed line II in Figure 3) the piezo voltage decreases at an adjustable rate. In other words, the pushing rod withdraws, and the gold–gold contact is formed again. Once the detected current reaches a preset “high limit” (typically 10 *G*_0_; dashed line III in Figure 3), the voltage ramp for the piezo stack is paused for up to 0.5 s, and a new cycle starts following an identical protocol. The entire traces, as acquired during the opening and closing process, were recorded for further data analysis.

The initial position for every opening/closing cycle may change due to changes in the gold–gold contact geometry, especially in the beginning of the experiments. However, as soon the voltage for the piezo stack output approachs one of the limits (lower limit: 0 V; higher limit: 50 V), the piezo stack is reset to a neutral position and the stepper motor is reactivated to form or to break gold–gold contacts. After such a “pre-conditioning period”, which typically lasts up to 30 min for a newly started experiment, no further resetting is needed.

The distance between the two gold electrodes in the MCBJ setup is calibrated with the assumption that the tunneling decay is identical to that in a STM-BJ setup under the same experimental conditions. Conductance–distance traces representing a well-defined tunneling response, e.g., without molecular plateaus, were recorded in a STM-BJ and in a MCBJ configuration. Subsequently the decay constant (log[∆*G/G*_0_]/∆z = 5.5 nm^−1^) of the STM-BJ experiments was chosen to scale the traces acquired in the MCBJ setup.

### Sample preparation

For MCBJ experiments, the following sample preparation protocol was applied: The sample templates were spring steel sheets (30 mm × 10 mm with 0.2 mm thickness), which were cleaned in boiling 25% nitric acid and Milli-Q water, and dried in a stream of argon. A gold wire of 100 μm in diameter was subsequently fixed on these sheets with two drops of preheated epoxy (40 °C, mixture of 100 STYCAST 2850 FT epoxy resin with catalyst 9; LakeShore, Westerville, OH). The distance between the two drops of epoxy glue was adjusted to be less than 500 μm. Next, the sample was conditioned overnight at 60 °C for epoxy polymerization. The freely suspended part of the wire was notched with a scalpel blade under an optical microscope to fabricate a constriction point. The as-prepared sample sheets were cleaned in boiling Milli-Q water for 15 min, rinsed with isopropanol and dried with argon before each experiment.

The Kel-F liquid cell including its cover, Kalrez O-ring and Teflon tubes for argon purging and solution exchange were cleaned in three alternating boiling cycles in 25% nitric acid and Milli-Q water to remove absorbed contaminants.

The sample sheet was first mounted on the sample holder of the MCBJ setup. Subsequently, the liquid cell was installed on top of the sample with a Kalrez O-ring attached to prevent leakage of the solution. The closed liquid cell was flushed with argon through an inert-gas cycling system to remove oxygen, and then the solution containing the test molecule was pumped into the liquid cell through a triple valve. The last step was repeated three times to reduce contaminations. Subsequently, the input and output valves for solution exchange and gas purging were closed, and the experiment started.

### STM-BJ experiment

Basic principles of the STM-BJ experiment, data analysis and sample preparation were described previously [16,33].

### Organic synthesis

The synthesis of the antraquinone-based cross-conjugated wire **AQ** followed a method reported previously [55]. Details on the synthesis of the anthracene-based linearly conjugated wire **AC** and of the molecular wire with broken symmetry **AH** will be communicated elsewhere [14,56]. The three dithiol-terminated molecular wires were synthesized with acetyl-protecting groups. Careful MCBJ and STM-BJ screening experiments with **AC** indicated that a high yield of single-molecule junctions is obtained in the absence as well as in the presence of in situ deprotecting agents, such as tetrabutyl ammonium hydroxide or triethylamine. As a consequence, and to keep the number of different species in the sample solution to a minimum [57], we performed the subsequent experiments with the acetyl-protected derivatives in the sample solution without implementing an additional deprotection step.

## Results and Discussion

### Conductance–distance measurements

#### Stretching traces

**Figure 4:**
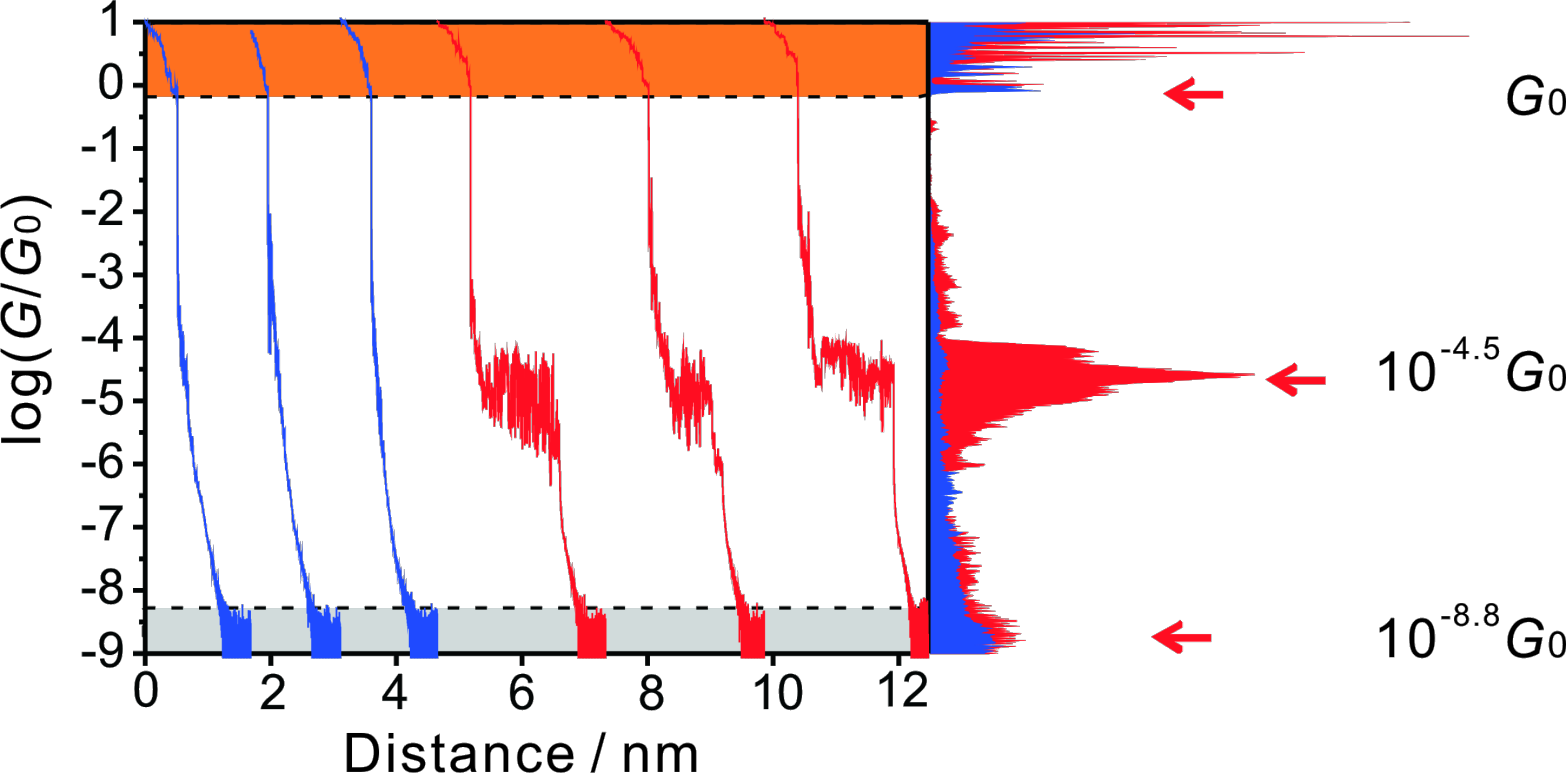
Individual conductance–distance traces and histogram constructed from these sets of three traces for **AC** at a bias voltage *V*_bias_ = 0.10 V in THF/decane (v:v = 1:4) under Ar atmosphere. The most probable conductance is indicated.

The measurements of conductance–distance traces in the MCBJ set up were carried out with 50 nm·s^−1^ as the typical rate for the movement of the pushing rod in the breaking process. This value translates into an approximate lateral movement between the two gold leads of about 1 nm. All data shown in the following sections and used for the analysis represent opening traces, which were recorded after breaking a gold–gold contact. Figure 4 shows six typical examples of individual traces in a log-conductance versus distance representation for the anthracene-based linear molecular wire **AC**. All curves start with characteristic steps and plateaus in the region between 10 and 1 *G*_o_ (orange part), representing the breaking of gold–gold atomic contacts. The last step is observed around 1 *G*_0_. After the gold–gold monatomic contact is broken, the two “separated” gold electrodes snap back and a nanogap is created with typical conductances ranging between 10^−2^ *G*_0_ and 10^−4^ *G*_0_. The snap-back process is too fast to be recorded with better resolution. At lower conductances we observed two distinctly different types of traces, those without molecular plateaus (blue curves, around 67 % of all data recorded) and those with molecular plateaus (red curves, around 33 % of all data recorded) in the range of 10^−4^ to 10^−5^ *G*_0_. The noise level is reached below 10^−8.2^ *G*_0_, which provides a wide window of over eight orders of magnitude for the single-molecule conductance measurements.

The blue traces in Figure 4 represent a tunneling response between the two broken gold leads through the solution without the formation of a molecular junction. These data were chosen for distance calibration. The red curves in Figure 4 indicate the successful formation of gold|**AC**|gold molecular junctions with a characteristic plateau. The three representative individual traces reveal a single plateau conductance at around 10^−4.5^ *G*_0_, which indicates the formation of a single-molecule junction between the two electrodes. The current noise is attributed to the thermal vibration of the molecular junction at room temperature. The conductance traces exhibit an abrupt decrease upon breaking of the molecular junction until the noise level is reached. The most probable conductance of the molecular junction was obtained by statistical analysis of the data. The resulting histogram, as constructed from the three red traces, is plotted in the right panel of Figure 4. The graph reveals a sharp and clear conductance peak at 10^−4.5^ *G*_0_, which is equal to 2.5 nS, the most probable single-molecular junction conductance of **AC** from a limited data set of three individual traces. (Note that the complete, statistically significant analysis is reported below in the section "Comparative conductance measurements of AC with AQ and AH"). Applying the same analysis method to the blue traces did not lead to any clear feature between 10^−1^ *G*_0_ and 10^−8^ *G*_0_, which supports the assignment of the two types of traces.

### Continuous current–voltage (*I–V*) measurement

#### *I–V* curves in the stretching process

**Figure 5:**
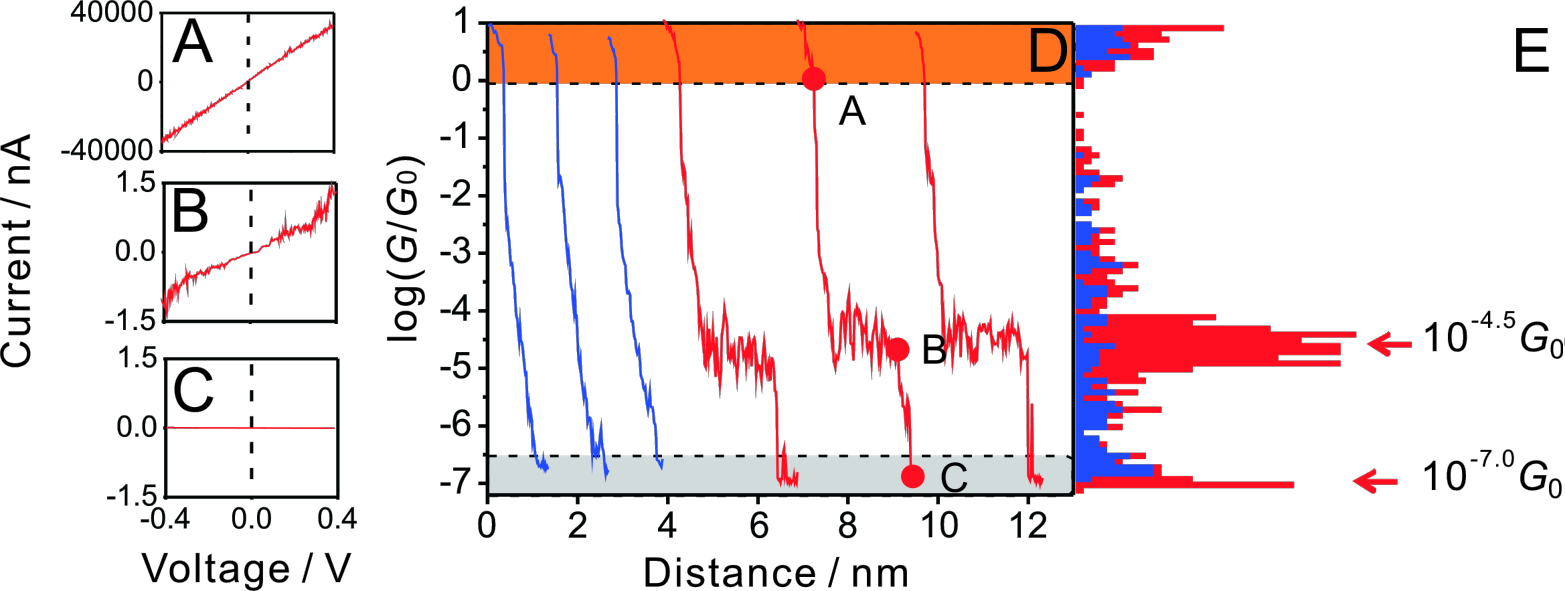
(A–C) Individual current–voltage curves of (A) a gold–gold contact, (B) a gold|**AC**|gold molecular junction, and (C) tunneling response through the solvent upon approaching the noise level. (D) Traces constructed from the slopes of individual *I–V* curves as recorded simultaneously during a slow stretching half cycle, with (red traces) and without (blue traces) the formation of a molecular junction. The red circles indicate the positions where the *I–V* curves shown in panels (A) to (C) were recorded. (E) Conductance histogram as constructed from the data points plotted in panel D. Other conditions: 0.1 mM in THF/decane (v:v = 1:4), Ar atmosphere.

The high mechanical stability of the MCBJ setup provides a unique platform to create stable gold|molecule|gold junctions with a lifetime of several seconds. For *I–V* measurements we controlled the opening and closing cycles by slowly moving the pushing rod at a rate of 0.5 nm·s^−1^ and we swept simultaneously the bias voltage *V*_bias_ from −0.4 V to +0.4 V at a rate of 25 V·s^−1^ at various positions. This approach resulted in a set of *I–V* curves spanning a range of conductance during a single opening and closing cycle, which correspond variously to the Au–Au contacts (Figure 5A), the formation of molecular junctions (Figure 5B), the tunneling through the solvent and, finally, the approach to the noise level (Figure 5C). *I–V* traces of the gold–gold contacts are linear, and represent ohmic characteristics, whereas *I–V* curves of the molecular junctions are nonlinear. They provide an important test platform to estimate the relative positions of molecular levels and the Fermi levels of the leads, based on a comparison with ab initio transport calculations and the corresponding transmission curves [58].

For comparison with the conductance–distance measurements shown in Figure 4, we calculated the slopes of the linear parts (typically in the range between −0.30 to 0.30 V) of individual *I–V* curves at different stages of the stretching process. We emphasize that each data point represents the conductance extracted from one *I–V* curve in the zero-bias limit. Initially (orange area in Figure 5D), all *I–V* curves exhibit the conductance of gold–gold contacts. Once this contact is broken, either one of two families of curves is observed. The blue traces in Figure 5D represent tunneling through the solvent without the formation of a molecular junction. The dotted log(*G/G*_0_) versus distance traces are linear until the noise level is reached. The second type of curves (red traces in Figure 5D) showed well-developed molecular plateaus. Employing 0.5 nm·s^−1^ as the pulling rate to separate the two gold electrodes enables the acquisition of 30 to 40 individual *I–V* curves in the conductance range of **AC** molecular junctions around 10^−4.5^ *G*_0_ during a single stretching trace. Data points below 10^−5^ *G*_0_ represent tunneling through the solvent and, finally, the approach to the noise level (grey region in Figure 5D). The statistical analysis, based on counting the number of data points per conductance interval in each individual trace, leads to the construction of the conductance histograms. The graph in Figure 5E shows a well-resolved maximum located at 10^−4.5^ *G*_0_, despite the limited number of data points (ca. 200 from three traces). This value represents the most probable conductance of a gold|**AC**|gold single-molecule junction, and is in perfect agreement with the result of the continuous current–distance measurements (Figure 4). The coincidence demonstrates convincingly the reliability of both experimental approaches chosen.

#### Statistical analysis of *I–V* curves of molecular junctions

Thermal vibrations as well as switching events between different configurations and conductance states in a molecular junction require a careful statistical analysis of several thousands of individual traces to extract the “most probable” *I–V* characteristics of a certain molecule under a given set of experimental conditions. This approach is particularly important for single-molecule experiments at a solid/liquid interface at room temperature.

**Figure 6:**
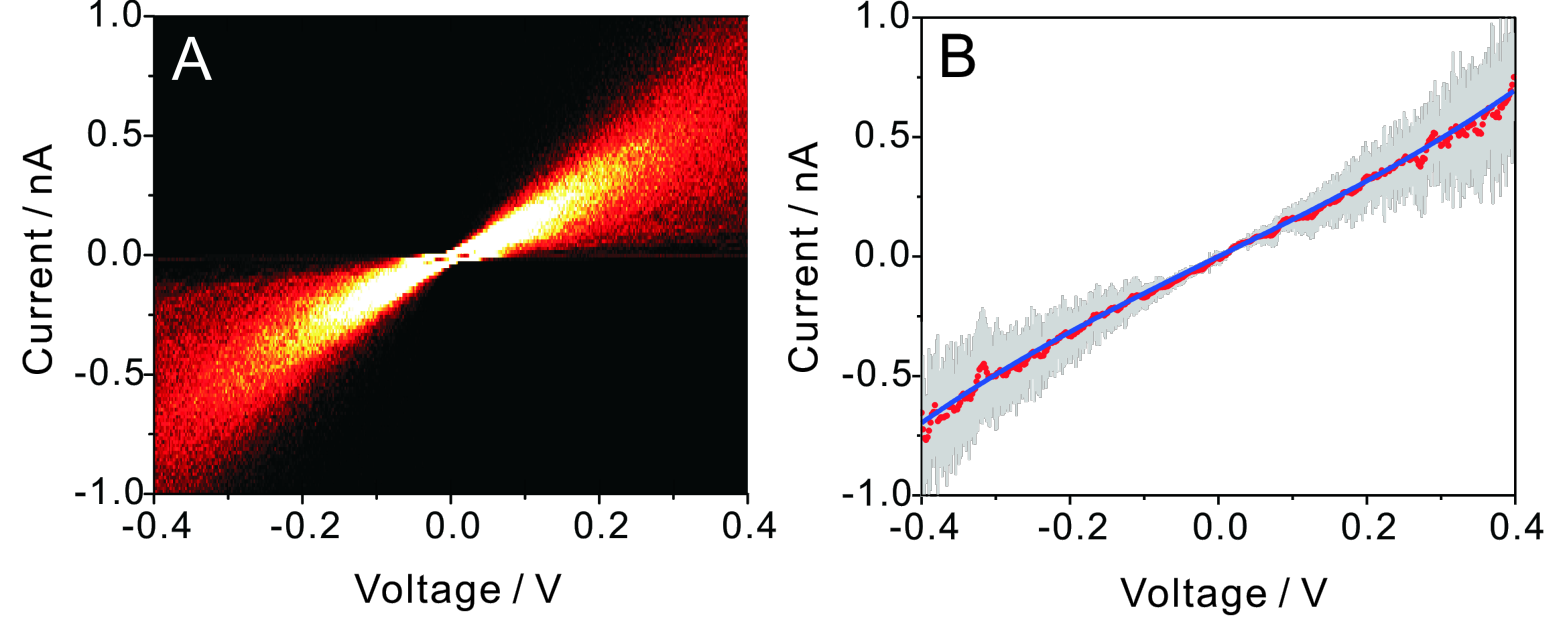
(A) 2-D *I–V* histogram constructed from 2500 individual traces recorded during a current–distance stretching experiment, stretching rate 0.5 nm·s^−1^, in the molecular junction region for 0.1 mM **AC** in THF/decane. (B) *I–V* “master curve” (red) and its standard deviation (error bar) obtained from Gaussian fits at constant bias voltages of the data plotted in panel A, and the corresponding model fitting (blue).

Figure 6A shows a 2-D histogram of 2500 *I–V* traces as recorded during individual stretching events in the region of molecular junction formation, i.e., from 10^−4.3^ to 10^−4.7^ *G*_0_. The color code demonstrates clearly the existence of preferred conductance states. Next we determined for each bias voltage *V*_bias_ the most probable current value and its standard deviation from a Gaussian fit. The choice of a Gaussian fit is justified because the distribution of the measured current preferentially originates from thermal vibration and electronic noise, which are both completely random processes.

Figure 6B illustrates the most probable *I–V* master curve of **AC** attached to two gold leads as obtained from the statistical analysis of individual traces in −0.40 V < *V*_Bias_ < 0.40 V. The shape of the *I–V* trace provides additional information for exploring the nature of the transport process. In a first approximation, we considered a single-level model in the low-bias limit and with the molecules coupled equally to the leads. We thus evaluated the experimentally observed *I–V* characteristics based on the following expression ([1] page 366, and [18]):

[1]
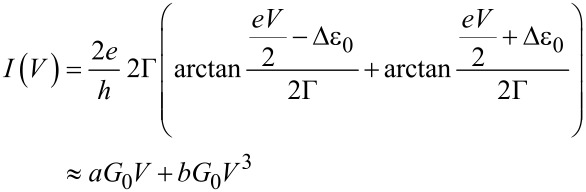


where Δε_0_ = ε_0_ − μ is the energy of a molecular level ε_0_ relative to the Fermi energy, and Γ is the resonance level width. The first expression is obtained by integrating a Lorentzian form for the transmission coefficient


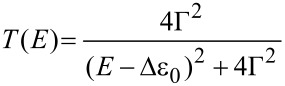


over a bias window 
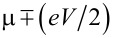
. The second expression, which is a cubic function with coefficients *a* and *b*, is expressed by Taylor expansion of the first, which yields


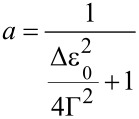


and


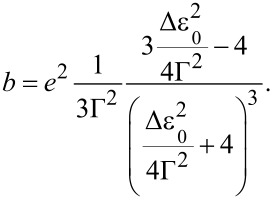


In practice, *a* and *b* are obtained by fitting the cubic function to the experimentally measured *I–V* curve, and Δε_0_ and Γ are then obtained from the inverse relations:

[2]
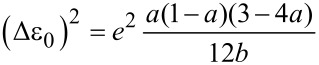


and

[3]
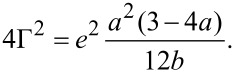


Fitting the model, as represented by Equation 1 to Equation 3, to the experimental *I–V* master curve of **AC** in the range of −0.40 V to 0.40 V provides an estimate of the two parameters as Δε_0_ = −0.53 eV (see text below) and Γ = 0.0012 eV. The negative sign is concluded from the following: Considering the Fermi energy of gold as −5.0 eV, we estimated −5.53 eV as the position of the *E*_HOMO_ level. This result is in good agreement with AM1-RHF calculations, performed with Hyperchem Release 7.52, and the level alignment, based on UPS data, of a related **AQ**-type molecular wire, *E*_HOMO_(Hyperchem_corr_) = −5.74 eV [14]. The HOMO–LUMO gap is estimated at 2.90 eV from the onset of UV–vis spectra in CH_2_Cl_2_ [14]. Based on these data we conclude that transport through **AQ**-type molecular junctions is HOMO-dominated.

The coupling parameter Γ appears to be rather small as compared to those for other dithiole-terminated molecular wires attached to gold leads [1,18]. These deviations might be related to the simplicity of the model chosen.

### Comparison between *I–V* and conductance–distance measurements of AC by MCBJ and STM-BJ

**Figure 7:**
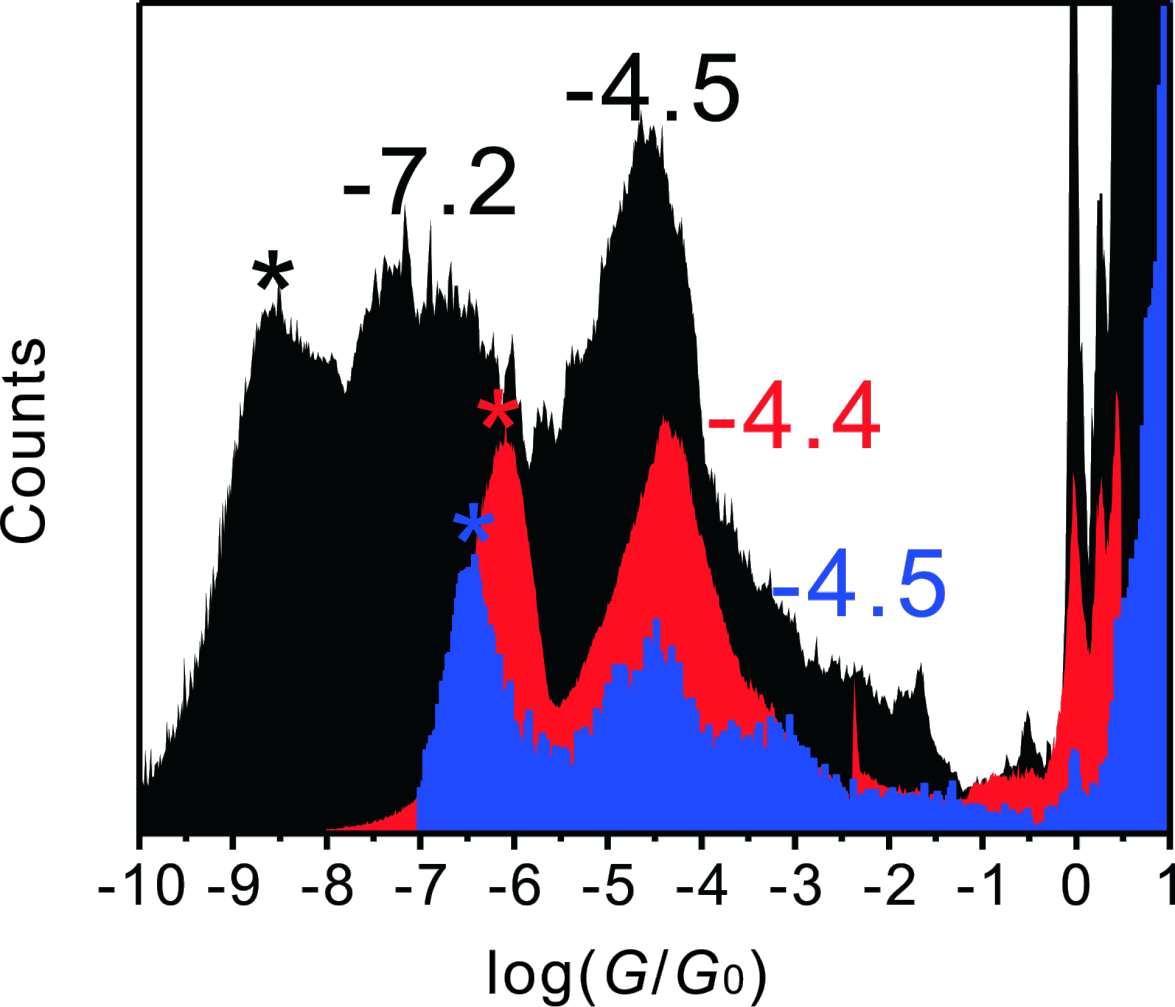
Conductance histograms of 0.1 mM **AC** in THF/decane (v:v = 1:4). Black: From 500 current–distance stretching curves in a MCBJ setup. Red: From 2000 current–distance stretching curves recorded in an STM-BJ experiment, with 0.10 V bias voltage. Blue: Based on 60,000 individual *I–V* traces measured simultaneously in the range of −0.40 to 0.40 V, in the molecular junction region of the above MCBJ experiment.

Figure 7 compares the conductance histogram of **AC**, constructed from the above *I–V* data (c.f. Figure 5 but now based on the analysis of 60,000 individual traces, which contain thousands of *I–V* curves in the molecular junction region, blue diagram in Figure 7), with that obtained from the analysis of 500 current–distance traces of the MCBJ setup (black diagram, without any data selection, c.f. also Figure 4). We also added the histogram (red diagram) that was obtained from the statistical analysis of 2000 individual traces acquired with our STM-BJ setup [15–16,33]. Both the red and the black graphs display clear peaks at 1 *G*_0_ and 10^−4.4^–10^−4.5^ *G*_0_, which are assigned to the breaking of a monatomic gold–gold contact and the single molecular junction conductance of **AC** trapped between two gold leads, respectively. The good agreement between the results of the three different experimental approaches indicates the reliability of the measurements as well as the independence of the single-molecule conductance values of the present system from the measurement techniques chosen.

Histograms based on the MCBJ and STM-BJ data are distinctly different with respect to the noise level. Due to different preamplifier designs and stabilization concepts, the noise level of the STM-BJ setup is reached at around 10^−6.0^ *G*_0_ (red asterisk in Figure 7), whereas the noise level of the MCBJ stage is significantly lower and appears to interfere with the junction response only below 10^−8.5^ *G*_0_ (black asterisk in Figure 7). In consequence, we were able to resolve an additional molecular junction-related feature around 10^−7.2^ *G*_0_ in the MCBJ transport experiments of **AC**, which is equal to 4.9 pS. We note that the new feature could not be detected in the STM-BJ experiments due to the sensitivity limitations.

The conductance histogram based on the statistical analysis of *I–V* traces (blue diagram in Figure 7) was constructed from 60,000 individual curves, which were recorded simultaneously with the 500 stretching traces. The analysis revealed a clear molecular junction conductance peak of 10^−4.5^ *G*_0_, which is in good agreement with the most probable values as extracted from the MCBJ and STM-BJ conductance–distance measurements. However, the low conductance range (<10^−6.5^ *G*_0_) could not be monitored reliably due to the relatively slow response of the log *I–V* converter in the pA range. The *I–V* converter could not follow precisely enough the current change in the low-conductance range upon sweeping the bias voltage at a rate of 25 V·s^−1^.

### Comparative conductance measurements of AC with AQ and AH

**Figure 8:**
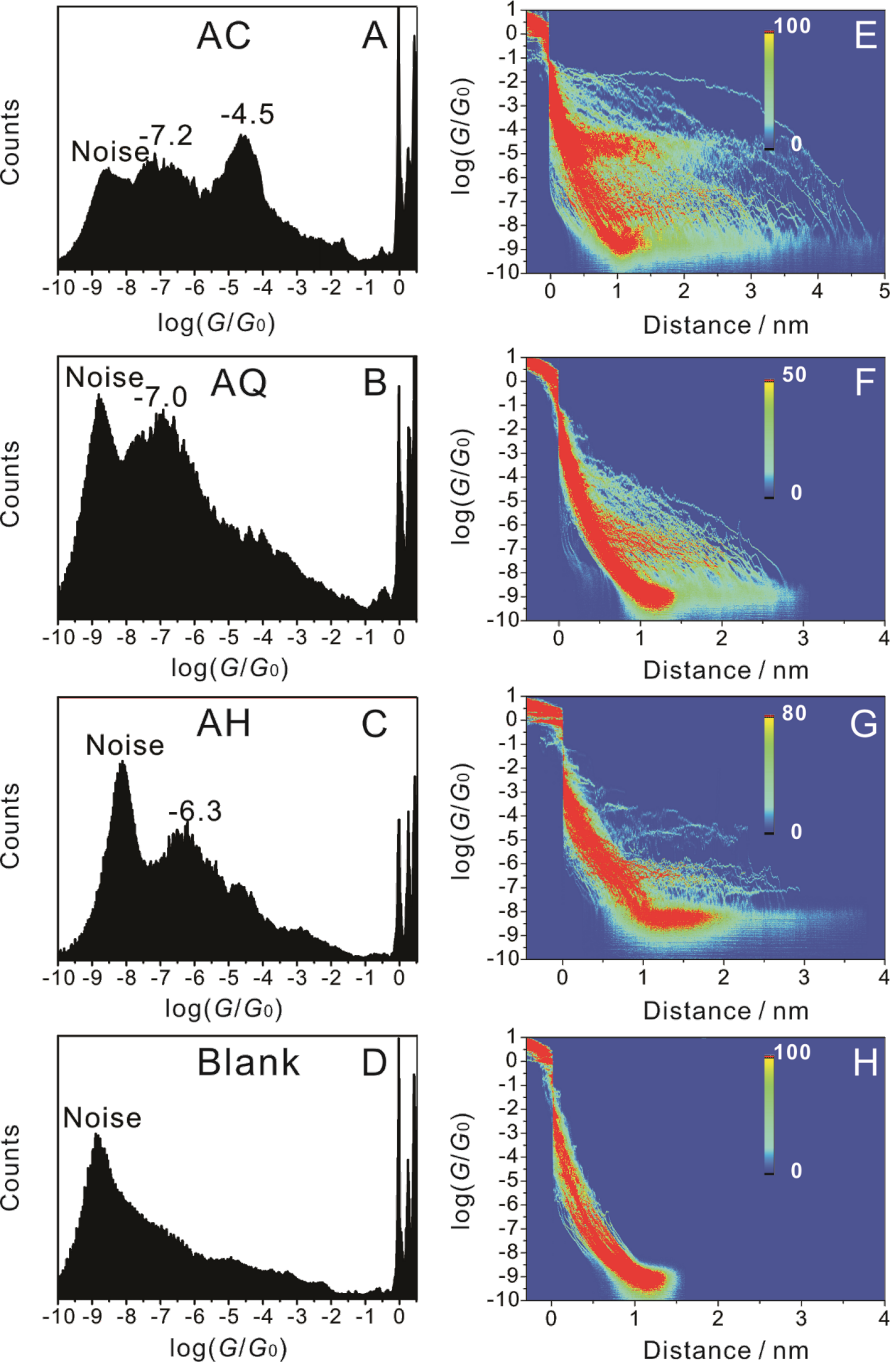
1-D conductance histograms and conductance–distance 2-D histograms constructed from 500 individual traces of **AC** (panels A and E), **AQ** (panels B and F), **AH** (panels C and G) and for the blank control experiment (panels D and H) in THF/decane (v:v = 1:4) under Ar atmosphere at 0.10 V bias voltage in the MCBJ setup. The molecule concentration was 0.1 mM, and the stretching rate was around 1 nm**·**s^-1^.

Figure 8 shows 1-D conductance histograms and 2-D conductance–distance histograms of **AC**, **AQ**, **AH** and, for comparison, also the target-molecule-free THF/decane solution, as obtained in a series of MCBJ measurements. All experiments were carried out under identical conditions and analyzed with the strategies introduced above. We note that the histograms constructed for the blank control experiment (Figure 8D and Figure 8H) do not show any significant conductance peaks, except the one attributed to the breaking of the monatomic gold–gold contact around *G*_0_ and the feature at 10^−8.8^ *G*_0_. The latter represents the noise level. The slight increase of the baseline in the histograms results from contributions of the gap-modulated tunneling current, which originates from variations in the solvent conformation as well as from the “snap-back” distances of the gold–gold nanocontacts upon breaking the leads [59–60].

Figure 8B reveals a clear peak at 10^−7.0^ *G*_0_ (7.8 pS) in the conductance histogram of the cross-conjugated anthraquinone wire **AQ**. This feature is well separated from the noise level, which is located at 10^−8.8^ *G*_0_. The junction conductance of **AQ** is approximately 300 times lower than that of **AC**. This trend demonstrates that the cross-conjugated motif of **AQ** indeed gives rise to a lower conductance as compared to the linear-conjugated **AC**, which is in agreement with ab initio transport calculations predicting a destructive quantum interference present in **AQ**, but which is absent in **AC** molecular bridges [24]. From a technical point of view, the accessibility and reliability of the low conductance data for the **AQ** molecular junction also illustrates the high sensitivity of our new MCBJ setup.

Figure 8C shows the 1-D conductance histogram of the dihydroanthracene wire **AH** with a broken π-conjugation. The plot reveals one main feature at 10^−6.3^ *G*_0_ (39 pS) and a faint second feature around 10^−4.5^ *G*_0_ (2.5 nS), the latter being 5 times larger and close to the data reported for **AC**.

Complementary to the 1-D histograms we also constructed, based on the above individual conductance–distance traces, 2-D conductance–distance histograms [61]. In an attempt to define a common reference point for all of the conductance–distance, we selected the position where the current reaches 0.1 *G*_0_ to define the relative zero of the distance scale [33]. The color code in Figures 8E to Figure 8H is chosen such that the red areas indicate a higher data density at the respective conductance–distance point. In agreement with the 1-D plot of **AC** in Figure 8A, Figure 8E shows a clear and dominant molecular plateau around 10^−4.5^ *G*_0_ and a second, less dense patch of data points, around 10^−7.2^ *G*_0_ indicating a low conductance feature. The 2-D histogram of **AQ** reveals only one clear molecular feature, which is found around 10^−7.0^ *G*_0_ (Figure 8F). On the other hand, the 2-D histogram of **AH** (Figure 8G) mainly displays a molecular feature around 10^−6.3^ *G*_0_ but also a weak intensity patch at 10^−4.5^ *G*_0_, which coincide with the main peak and a weak secondary feature shown in the 1-D conductance histograms (Figure 8C). We comment that the overall evolution of the minority feature of **AH** is close to the main conductance peak of **AC**.

**Figure 9:**
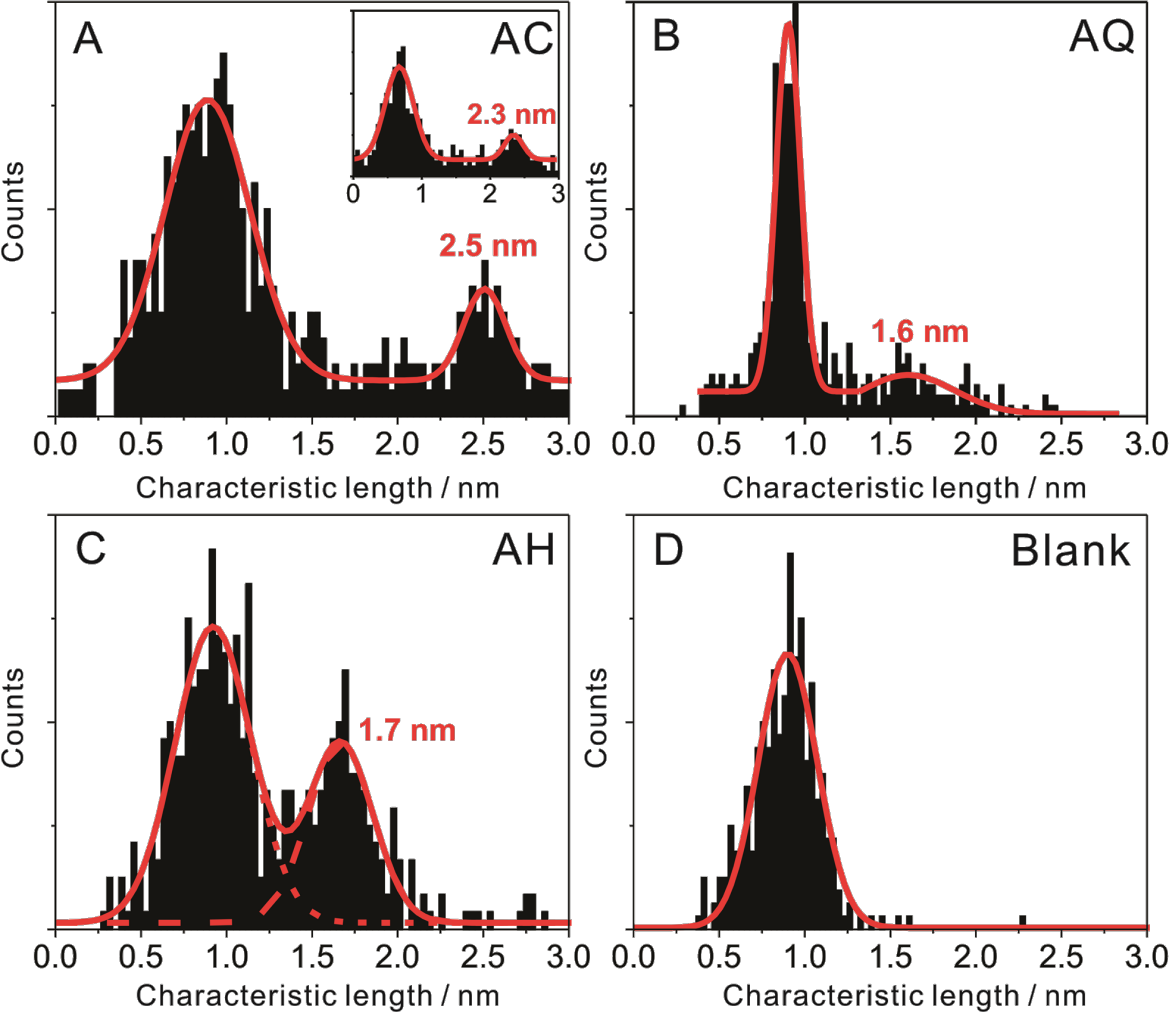
Plateau-length distributions (black) and Gaussian fits (red) of (A) **AC** (B) **AQ** (C) **AH** and (D) the blank control system, as constructed from the data shown in Figure 8. The conductance range selected for the plateau length analysis ranges between 0.1 *G*_0_ and 10^−8^ *G*_0_. The inset in panel (A) represents the plateau length analysis of **AC** in a limited conductance range from 0.1 *G*_0_ to 10^−6^ *G*_0_ in order to extract the length of the main conductance plateau.

We further analyze the stretching distance from the breaking of gold–gold contacts until the noise level is reached (from 10^−1^ *G/G*_0_ to 10^−8 ^*G/G*_0_). We extracted the most probable stretching distance of breaking for the high conductance plateau of **AC** in the range of 10^−1^ *G/G*_0_ to 10^−6 ^*G/G*_0_ to explore further details of the low-conductance state. As illustrated in Figure 9A, we observed two, clearly separate peaks. The first peak, located around 1 nm, is assigned to a tunneling feature without the formation of a molecular junction (blue traces in Figure 4). The second peak, which evolves at longer stretching distances, results from the formation of a molecular junction and reflects properties of a true molecular plateau (red traces in Figure 4). The most probable “relative” stretching distance at which the **AC** molecular junction breaks is 2.5 nm, while the most probable stretching distance up to the end of the high-conductance molecular plateau is obtained as 2.3 nm (inset in Figure 9A). The difference of 0.2 nm is attributed to a low-conductance feature. The most probable “real” plateau length of the gold|**AC**|gold junctions is estimated at 3 nm by adding the “snap-back” distance of 0.65 nm [17,59–60] resulting from the breaking of the monatomic gold–gold contact. This value is slightly higher than the molecular length of **AC** (2.7 nm). We propose that the strong gold–thiole bond leads to the “pulling-out” of surface gold atoms just before the breaking of the molecular junction. The low-conductance feature is attributed to π-stacking interactions between two molecules attached only at one end of the leads [44,62]. For a more detailed and critical discussion of possible junction geometries and molecular mechanisms of junction breaking, we refer to our forthcoming papers [14,17].

Figure 9B and Figure 9C show that the “relative” plateau length of **AQ** amounts to 1.6 nm, while **AH** is estimated at 1.7 nm. After correction with the snap-back distance one obtains 2.25 nm and 2.35 nm. Both values are smaller than the molecular length, which indicates that most of the molecular junctions break before they are completely elongated, which is distinctly different behaviour compared to **AC**. We note that the maximum in the adsorbate-free control experiment at around 0.9 nm (Figure 9D) results from tunneling and noise contributions, and is not related to the formation of gold|molecule|gold junctions. Introducing a “relative” distance of 1.25 nm as a threshold for the identification of a molecular junction, we calculated the junction formation probability from the plateau-length analysis and obtained the following values: 33% out of all traces for **AC**, 32% for **AH** and 14% for **AQ**. Clearly, the molecular structure of each of the three OPE-type species influences the bonding of the molecule to the gold-electrodes as well as the formation probability of the junction.

**Figure 10:**
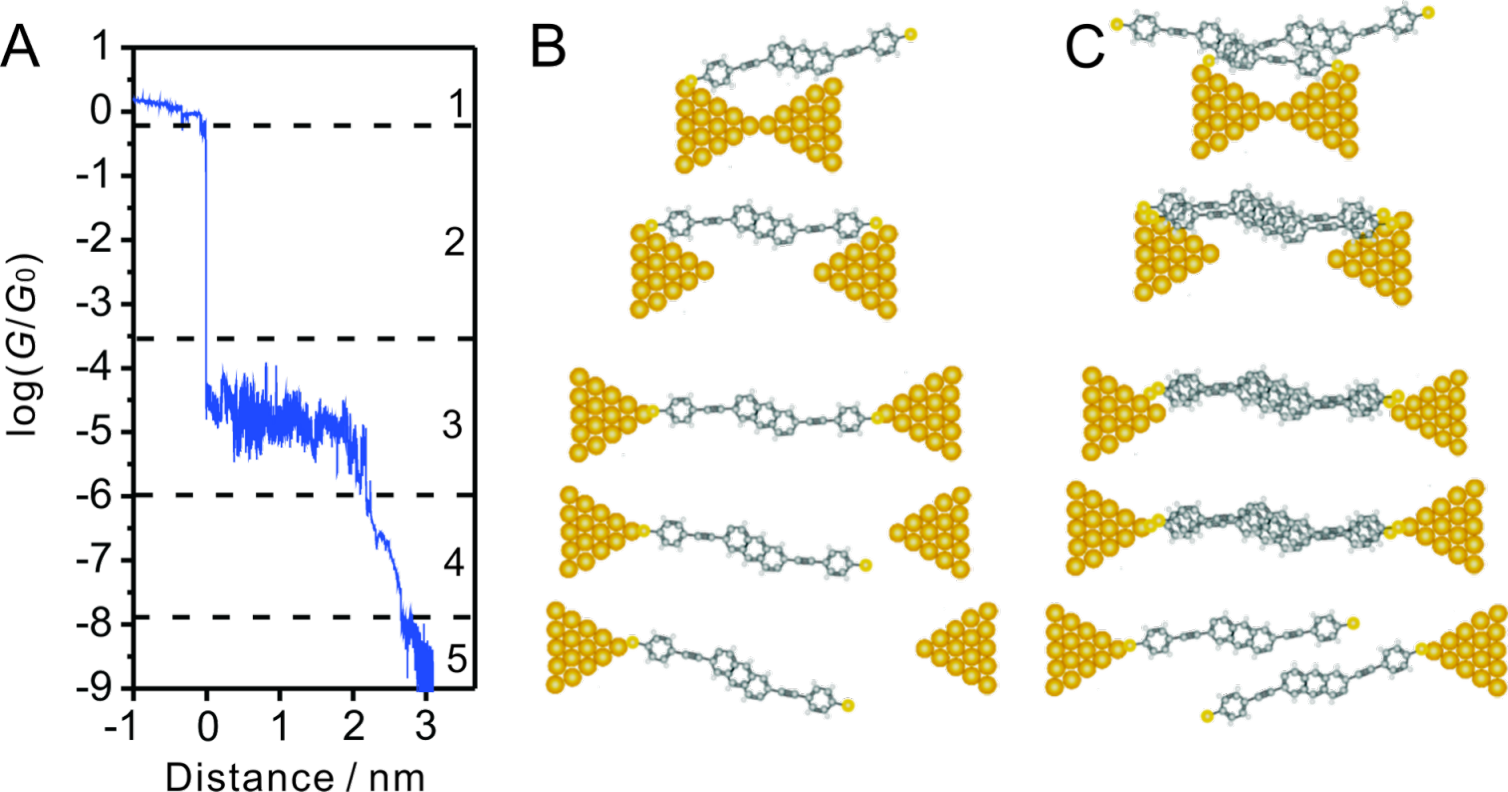
Model of the breaking process of the Au|**AC**|Au junction. (A) A typical trace with labels indicating various stages of the process. (B) Model of the formation and breaking of a single-molecule junction. (C) Model for a scenario involving the stacking of two molecules.

Based on the analysis above, we suggest the following as the most probable scenario to explain the features of a stretching trace in the high conductance regime of **AC**: The gold leads retract (“snap-back”) upon breaking of an atomic gold–gold contact (configurations 1 and 2 in Figure 10B). Subsequently, the **AC** molecule “slides” into the junction and connects finally to both gold electrodes. The conductance changes slightly upon further pulling [63] until the molecule is completely trapped (configuration 3 in Figure 10), which leads to the most probable conductance value of 10^−4.5^ *G*_0_ for a single gold|**AC**|gold junction. Further pulling causes an elongation of the Au–thiol bond until the junction breaks. The low-conductance feature is attributed to the formation of molecular stacks after breaking of the gold leads [44,62]. A tentative scenario is illustrated in Figure 10C. Both processes may occur sequentially if more than one molecule is trapped in the junction. This interpretation is based on experimentally observed “stacking” trends in single-molecule junctions formed by dithiolated or monothiolated OPE-type molecules attached to two adjacent gold leads [44,62].

We also note that the high conductance value of a gold|**AC**|gold junction follows the trend ln *G* = ln *A* – β·*L*, with *L* as molecular length, *A* = 10,819.6 nS and β = 3.4 nm^−1^ as experimentally observed for the length dependence of a family of dithiol-terminated OPE-molecules [14]. β is the tunneling decay constant, which is approximately 2.5 times smaller than typical values for aliphatic molecular wires.

**AQ** and **AH** form only one type of molecular junction, which we attribute to the “high” conductance type. However, the two most probable values obtained do not follow the above trend for the conductance versus length dependence of unsubstituted dithiolated OPE molecules. Both values, 10^−7.0^ *G*_0_ for the cross-conjugated anthraqinone **AQ** and 10^−6.3^ *G*_0_ for the dihydroanthracene **AH** are significantly smaller than predicted from this correlation. The data of Figure 8 reveal the following trend in single-junction conductance: **AC** > **AH** > **AQ** for this series of molecules with identical lengths. This trend thus clearly shows the influence of the π-conjugation pattern on the single-molecule conductance. The even lower conductance of the cross-conjugated molecule **AQ** as compared to the dihydroanthracene **AH** wire is attributed to a destructive quantum interference in the **AQ**-type junction [24,56]. Complementary data from single-molecule *I–V* traces were only accessible for **AC** (section "Continuous current–voltage (*I*–*V*) measurement"), and not for **AQ** and **AH**. The rather low junction-conductance characteristics of these two molecules as recorded simultaneously during single stretching traces were too much distorted by the onset of instrumental noise.

Furthermore, the most probable molecular junction lengths of **AQ** and **AH** are smaller than the molecular length indicating that the junctions breaks more frequently before the molecule assumes an extended atop–atop configuration between the two ends of the gold leads. A peculiarity appears in the form of a weak conductance feature observed for **AH** around 10^−4.5^ *G*_0_ (Figure 8C and Figure 8H). The similarity to the main conductance feature of **AC** (Figure 8A and Figure 8E) suggests that the second conductance peak of **AH** may come from the partial oxidation of **AH** to **AC**.

Finally, we notice that the same sequence of conductance values as found in the MCBJ-experiments (**AC** > **AQ** > **AH**) was also observed in current-probe atomic force microscopy (CP–AFM) [64] and EGaIn studies [65] of these three molecules. However, the absolute conductance values were about two (CP–AFM) to five (EGaIn) orders of magnitude larger. This difference is attributed to the contact area in the CP–AFM and EGaln setups. The conductance data acquired in such configurations represent the integral sum over parallel molecular junctions in asymmetric contact geometries, which is distinctly different from the single-molecule data reported in this paper. Furthermore, the number of bridging molecules, which contribute to the measured charge-transfer characteristics in the large-area molecular junctions, is not directly accessible, thus preventing the downscaling to an “effective” single-molecule-junction response.

## Conclusion

We described in this paper the construction of a mechanically controlled break-junction setup (MCBJ) equipped with a highly sensitive log *I–V* converter to measure ultralow conductances of molecular rods formed between two gold leads. In particular, we carried out quantitative single-molecule conductance experiments on linearly conjugated, cross-conjugated, and broken-conjugated examples of dithiolated molecules of the OPE family. The current sensitivity of the setup reaches down to 10 fA. Our experiments demonstrate that the conductance of the linearly conjugated molecule **AC** is several hundred times higher than that of the broken π-conjugated molecule **AH**, and the conductance of **AH** is about five times higher than that of the cross-conjugated molecule **AQ**. The latter result is attributed to destructive quantum interference present in the **AQ** molecular bridge [24]. All dithiolated molecules are of similar length (~2.6 nm), but only **AC** appears to be capable of forming a large number of fully extended gold|molecule|gold junctions. The other two molecules **AQ** and **AH** form junctions that break before reaching full extension.

These experimentally observed trends in the values of the single-molecule conductances as well as in the stability of the respective molecular junctions reveal the key role of π-conjugation in the charge transport through rigid-rod OPE-type single-molecule junctions. Moreover, the good agreement between the different measurement approaches employed in this paper (current–distance and current–voltage traces from MCBJ and STM-BJ) confirm the reliability of our measurements. The observation of similar trends in the main conductance values discovered in single-molecule (MCBJ, STM-BJ) and parallel-molecule junction experiments (CP–AFM, EGaln junctions) confirms the complementarity of the various experimental platforms, in both their similarities as well as their distinct differences.
